# Reassessing hierarchical correspondences between brain and deep networks through direct interface

**DOI:** 10.1126/sciadv.abm2219

**Published:** 2022-07-13

**Authors:** Nicholas J. Sexton, Bradley C. Love

**Affiliations:** ^1^ Department of Experimental Psychology, University College London, London, UK.; ^2^ The Alan Turing Institute, London, UK

## Abstract

Functional correspondences between deep convolutional neural networks (DCNNs) and the mammalian visual system support a hierarchical account in which successive stages of processing contain ever higher-level information. However, these correspondences between brain and model activity involve shared, not task-relevant, variance. We propose a stricter account of correspondence: If a DCNN layer corresponds to a brain region, then replacing model activity with brain activity should successfully drive the DCNN’s object recognition decision. Using this approach on three datasets, we found that all regions along the ventral visual stream best corresponded with later model layers, indicating that all stages of processing contained higher-level information about object category. Time course analyses suggest that long-range recurrent connections transmit object class information from late to early visual areas.

## Teaser

Model-brain correspondences established by substituting parts of models with brain activity explain brain function.

## INTRODUCTION

Despite some shortcomings (*1*, *2*), deep convolutional neural networks (DCNNs) have emerged as the best candidate models for the mammalian visual system. These models take photographic stimuli as input and, after traversing multiple layers consisting of millions of connection weights, output a class or category label. Weights are trained on large datasets consisting of natural images and corresponding labels.

**Fig. 1.**
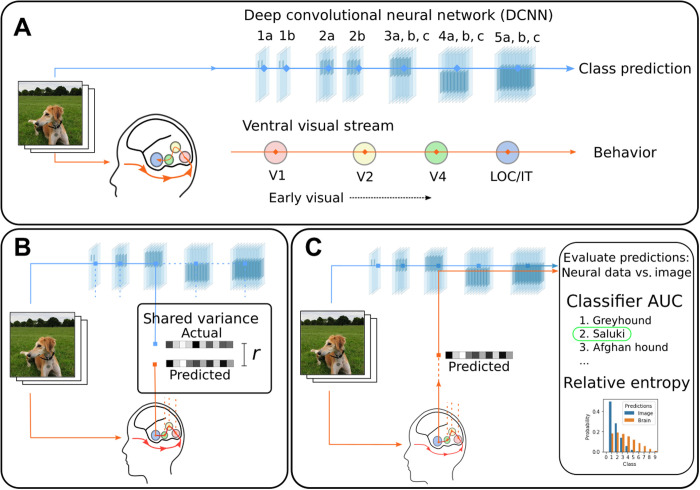
DCNNs trained on large naturalistic image datasets have emerged as leading models of the mammalian ventral visual stream. (**A**) Typically, processing in DCNNs is hierarchical starting with the stimulus and proceeding across successive layers as higher-level information is extracted, culminating in predicting the class label (*14*). Numerous analyses (*3*–*5*) based on shared variance suggest that the brain follows related principles with an early-to-early and late-to-late pattern of correspondence between the ventral visual stream and DCNN layers. LOC, lateral occipital complex; IT, inferotemporal cortex. (**B**) These shared variance correspondences are evaluated locally, typically involving one brain region and one model layer, with no recourse to behavior (i.e., the object recognition decision). (**C**) We propose a stronger test of correspondence based on task-relevant variance (i.e., activity relevant to the model’s task). If a model layer and brain region correspond, then model activity replaced with brain activity should drive the DCNN to an appropriate output (i.e., decision). The quality of correspondence is evaluated by comparing DCNN performance when driven by a stimulus image versus interfaced with brain activity. AUC, area under the receiver-operator characteristic (ROC) curve. Photo credit: N. J. Sexton, University College London.

The deep learning revolution in neuroscience began when layers of DCNNs were related to regions along the ventral visual stream in an early-to-early and late-to-late pattern of correspondence between brain regions and model layers (Fig. 1A) (*3*–*5*). This correspondence supported the view that the ventral stream is a hierarchy in which ever more complex features and higher-level information are encoded as one moves from early visual areas like V1 or V4 to inferotemporal (IT) cortex (*6*–*8*).

However, the conceptual foundations of what constitutes a satisfying correspondence deserve scrutiny. Although the field uses a variety of methods to assess correspondence (*4*, *5*, *9*), they all focus on some notion of fit or correlation between model and brain measures. In essence, all of these approaches measure the variance shared between brain and model activity (Fig. 1B). This hidden assumption may be problematic. Much of cortex-wide neural variance does not relate to the task of interest (*10*) and may covary with but not drive behavior. Correspondences established by correlation alone do not necessitate that model layers and brain regions play the same functional role in the overall computation. Just as correlation does imply causation, correlation does not imply correspondence.

We propose a stronger test for evaluating how brain-like a model is. If, as is frequently claimed (*3*–*5*), a specific layer in a DCNN corresponds to a brain region, then it should be possible to substitute the activations on that layer with the corresponding brain activity and drive the DCNN to an appropriate output [cf. (*11*–*13*) and Fig. 1C]. For example, if we take V4 activity from a monkey viewing an image of a car and interface that brain activity with an intermediate DCNN layer hypothesized to correspond to V4, then the DCNN should respond “car” absent any image input. How well the DCNN performs when directly interfaced (through a simple linear mapping) (see Materials and Methods) with the brain provides a strong test of how well the interfaced brain region corresponds to that layer of the DCNN. In the direct interface approach, shared variance is not assessed. Rather than rely on statistical measures of correspondence, we assess brain-model correspondences in the context of the computational model performing a task of interest. In that sense, we are only concerned with the task-relevant variance, which is the variance that can drive the model to perform its task.

## RESULTS

### Driving model response with brain activity

We interfaced a pretrained DCNN, VGG-16 (*14*), with data from two human brain imaging studies (*15*, *16*) and a macaque monkey study (*17*). All three studies involved viewing complex images. For a chosen model layer and brain region, we calculated a linear mapping from brain to model activity by presenting the same images to the model for which we had neural recordings (Fig. 1C). This simple linear mapping is a translation between brain and model activity. We evaluated the quality of this translation by considering held-out images and brain data that were not used in calculating the linear mapping (Materials and Methods).

**Fig. 2.**
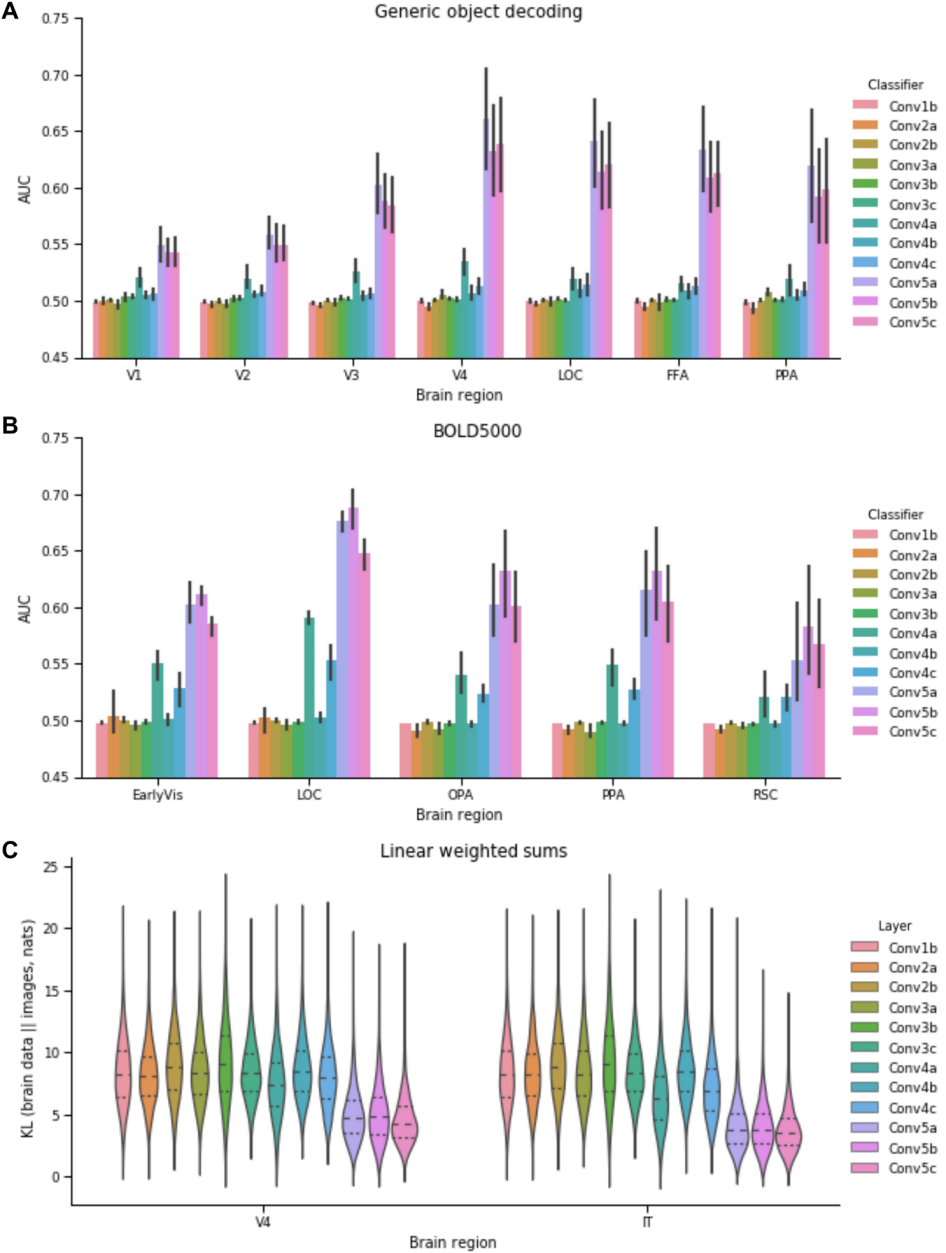
Results from interfacing neural data with VGG-16, a DCNN. Using the method shown in Fig. 1C, brain activity is directly inputted to a model layer to assess correspondence between a brain region and a model layer. (**A**) For this human fMRI study (*16*), all brain areas drive DCNN object recognition performance to above chance levels. The performance is best for all brain areas when interfaced with later model layers. (**B**) The same pattern of results is found for a second human fMRI study (*15*). OPA, occipital place area; PPA, parahippocampal place area; RSC, retrosplenial cortex. (**C**) In a third study, KL divergence is used (see Results and the Supplementary Materials) to measure the degree of correspondence for when the DCNN is driven by image input versus multiunit recordings from macaque monkeys (*17*). For KL divergence, lower values indicate better correspondence. Once again, all regions best correspond to later network areas. These three analyses indicate that higher-level visual information is present at all stages along the ventral visual stream.

Notably, for the two functional magnetic resonance imaging (fMRI) studies (Fig. 2, A and B), the DCNN was most accurate at classifying previously unseen images when the brain activity across regions (both early and late along the ventral stream) was interfaced with later model layers. In contrast to previous analyses that focused on shared variance, we did not find the early-to-early and late-to-late pattern of correspondence. Even primary visual cortex, V1, best drove the DCNN when interfaced with an advanced layer. For comparison, classifiers commonly used to decode information from fMRI data through multivariate pattern analysis (MVPA) were at chance levels (fig. S2), which highlights the useful constraints captured in the pretrained DCNN. After training on a million naturalistic images, the DCNN developed representations that paralleled those of the ventral stream, which made decoding object class possible by way of a linear mapping from the brain activity to an advanced DCNN layer.

The interpretation is that all brain regions contain advanced object recognition information, which conflicts with strict hierarchical views of the ventral visual stream. The hierarchical view implies an early-to-early and late-to-late pattern of correspondence, which we did not observe using our direct interface approach.

To rule out any alternative explanation based on the indirect nature of fMRI recordings, we considered a third study consisting of direct multiunit recording of spiking neurons implanted in the ventral visual stream of macaque monkeys (*17*). These monkeys were shown images that did not readily align with the pretrained DCNN’s class labels, so we evaluated neural translation performance by comparing the outputs of the DCNN when its input was a study image versus when a DCNN layer was driven by brain data elicited by the same image. For the distance measure, KL (Kullback Leibler) divergence, lower values imply a better translation between brain and model activity. As in the fMRI studies, both relatively early regions (i.e., V4) and late regions (i.e., IT) best translated to later DCNN layers (Fig. 2C). Note that while V4 is intermediate in ventral stream, we subsequently refer to it as “early” as relative to IT and for consistency with other datasets.

Across three diverse studies, we found a remarkably consistent pattern that strongly diverged from previous analyses—both early and late regions along the ventral visual stream best corresponded (i.e., translated) to the late model layers. It is not that previous analyses were poorly conducted [see fig. S1 for a successful reanalysis of data (*17*) finding the early-to-early and late-to-late canonical pattern]. Rather, our novel analyses focused on task-relevant activity, i.e., variance that can drive behavior, provided a different view of the system than standard statistical analyses focused on shared variance. Integrating these two views suggests a nonhierarchical account of object recognition marked by long-range recurrence transmitting higher-level information to the earliest visual areas.

### Long-range recurrence as opposed to strict hierarchy

**Fig. 3.**
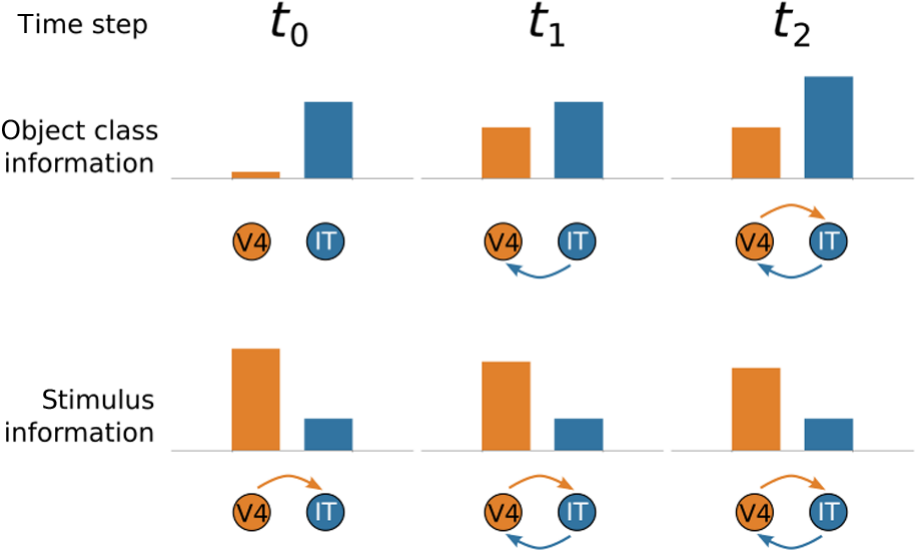
Hypothesized interactions between early (V4) and late (IT) regions along the ventral visual stream as processing unfolds. We hypothesize how stimulus and object-class information propagate between V4 and IT over time. At *t*_0_, the forward pass reaches IT from V4, with V4 activity reflecting low-level stimulus properties but little information about object class. At *t*_1_, object-class information from IT flows back to V4, increasing its task-relevant activity, which, in turn, influences IT at *t*_2_. Notice that later in processing, V4 reflects object class information, but most of its activity remains tied to bottom-up stimulus properties. These hypothesized interactions would reconcile our results (Fig. 2) based on task-relevant information with previous results based on shared variance.

One way to reconcile the existing literature based on shared variance with our analyses based on task-relevant activity is to propose that long-range connections from IT transmit higher-level information to early visual areas. Even if most variance in lower-level visual areas is attributable to stimulus-driven, bottom-up activity, most of the task-relevant information could be attributable to signals originating from IT (Fig. 3).

This view predicts specific patterns of Granger causality between early and late areas along the ventral visual stream. Do past values of one time series predict future values of the other? In terms of total spiking activity, lower-level areas should first cause activity in higher-level areas during the initial feed-forward pass, in which stimulus-driven activity propagates along the ventral visual stream. Later in processing, the causality should become reciprocal as top-down connections from IT affect firing rates in the lower-level areas, such as V4 (Fig. 3, bottom row). In contrast, Granger causality for task-relevant information should be first established from IT to V4 (i.e., the top-down signal) and only later in processing should recurrent activity lead to causality from V4 to IT (Fig. 3, top row). In this fashion, all areas are effectively “late” after long-range recurrent connections transmit information from IT to early visual areas along the ventral stream although most variance for these areas would be dominated by lower-level (bottom-up) stimulus information.

**Fig. 4.**
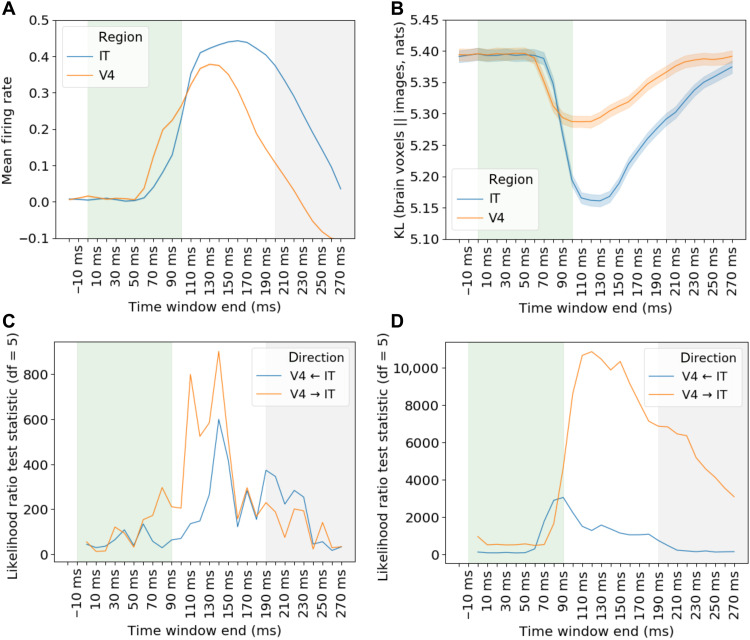
Analyses of monkey multiunit recordings time locked to stimulus presentation in 10-ms time bins. In the recordings (*17*), each visual stimulus was presented for 100 ms (shaded green) with 100 ms before the next (shaded gray). (**A**) Mean normalized spike counts for all electrodes for V4 and IT. (**B**) Task-relevant analysis (lower values imply closer correspondence with a late DCNN layer) shows that both V4 and IT can appropriately drive DCNN response (Fig. 1B), starting around 70 ms after stimulus onset. (**C**) Consistent with our long-range recurrence hypothesis (Fig. 3), Granger causal modeling indicates that, while V4 first drives IT in terms of raw firing rates (*V*4 → IT), (**D**) IT first drives V4 in terms of task-relevant information (V4 ← IT). These results are consistent with information about object category information (as assessed by interfacing with a late layer in a DCNN), first arising in IT and then feeding back to V4. At later time steps, Granger causality between V4 and IT becomes reciprocal (V4 ↔ IT) as the loop cycles.

We tested these predictions using the monkey multiunit spiking data (*17*) that has the temporal resolution to support the analyses. Images were presented one after the other, each visible for 100 ms, with a 100-ms period between stimuli. Figure 4A shows the mean firing rates (10-ms time bins) with activity in V4 increasing shortly before IT, consistent with stimulus-related activity first occurring in V4. Figure 4B revisits our previous analyses (Fig. 2C) but with spike counts binned into 10-ms intervals rather than aggregated over the entire trial. Even with only 10 ms of recordings, neural translation from V4 and IT to an advanced DCNN network layer minimizes KL divergence between model outputs arising from image input versus when driven by brain activity.

Turning to the key Granger causality analyses, we evaluated whether early ventral stream regions become more like late ventral stream regions over time due to recurrence (Fig. 3). As processing unfolded, we found mutual causality between the lower-level (V4) and higher-level (IT) areas for analyses conducted over spike counts (Fig. 4C) and for analyses on the KL divergence times series that assessed the ability of the brain regions to drive DCNN response (Fig. 4D).

Critically, the specific predictions of the long-range recurrence hypothesis were supported with V4 first driving IT (V4 → IT) for the analysis of spike counts but IT first driving V4 (V4 ← IT) for the task-relevant information analysis using the KL divergence time series (see Materials and Methods for details). These results are consistent with the stimulus-driven bottom-up activity proceeding from V4 to IT on an initial feed-forward pass through the ventral stream with actionable information about object recognition first arising in IT. Then, recurrent connections from IT to V4 make task-relevant information available to V4. As this loop is completed and cycles, both areas mutually influence one another with the impact of bottom-up stimulus information maintained throughout the process.

## DISCUSSION

Computational models can help infer the function of brain regions by linking model and brain activity. Multilayer models, such as DCNNs, are particularly promising in this regard because their layers can be systematically mapped to brain regions. The deep learning revolution in neuroscience began with analyses suggesting an early-to-early and late-to-late pattern of correspondence between DCNN layers and brain regions along the ventral visual stream during object recognition tasks (*3*–*5*).

However, as we have argued, correspondences based on shared variance should be treated with caution. To complement these approaches, we presented a test focused on task-relevant activity that directly interfaced neural recordings with a DCNN model. If a brain region corresponds functionally to a model layer, then the brain activity substituted for the model activity at that layer should drive the model to the same output as when an image stimulus is presented. Of course, models and brains speak different languages, so a translation between brain and model activity must first be learned, which, in our case, was accomplished by a linear transformation. Once the translation function is learned, novel brain data and images can be used to evaluate possible brain-model correspondences.

Our approach, which focuses on task-relevant activity within the overall computation, as opposed to shared variance (Fig. 1) uncovered a pattern of correspondences that markedly differed from the existing literature. We found that all brain regions, from the earliest to the latest of visual areas along the ventral stream, best corresponded to the later model layers. These results indicate that neural recordings in all regions contain higher-level information about object category even when most variance in a region is attributable to lower-level stimulus properties (Fig. 3).

To resolve this discrepancy between our analyses focused on task-relevant activity and those based on shared variance, we evaluated the hypothesis that long-range recurrence between higher-level brain regions, such as IT, influenced activity in lower-level areas like V4. Analyzing both firing rates of cells and information-level analyses using our brain-model interface approach, we found evidence that recurrent activity renders all areas functionally late as processing unfolds, even when most activity in some early visual regions is largely driven by bottom-up stimulus information. In this way, we integrate previous findings with our own and highlight how our method can be used to test hypotheses about information flow in the brain.

One concern is that our notable results may arise from some artifact. For instance, perhaps the data quality or dimensionality recordings from early visual regions were insufficient to drive the DCNN’s lower layers, which themselves are high dimensional. First, logically, this argument fails because claims of correspondence require high-quality data. For example, it would be imprudent to claim that a single noisy neuron functionally corresponds to an early network layer because such data are not functionally sufficient to carry the relevant state information, which we directly assess in our substitution approach. Second, in terms of network dimensionality, because we project from the brain to the model, rather than the other way around as in encoding approaches, differences in extrinsic network dimensionality are not critical as each DCNN unit is essentially fit by its own regression model (i.e., the covariance term is on the brain side, not the DCNN side).

A related critique is that simply being early is a disadvantage because errors in mapping from brain data to a network layer may be magnified across later processing stages. Again, this argument ignores the logic of our substitution approach in that sensitivity to perturbations at early layers implies that higher quality data are required to establish functional correspondences. Empirically, we observe the exact opposite when perturbing networks—the network shows attractor-like behavior, in which successive stages of processing remove added noise rather than amplify it (fig. S7). Last, we can observe correspondences to early network layers, such as when we use an image as the data source rather than brain data (fig. S6), which indicates that there is no inherent bias in the substitution approach.

Our approach, which considers task-relevant activity or variance, may help resolve conflicting interpretations on the function of brain regions. For example, the fusiform face area (FFA) responds selectively for faces, but its wider functional role in object recognition has been the subject of extensive debate (*18*). Here, we show that interfacing FFA into the late model layers drives object recognition comparably to the lateral occipital complex (Fig. 2B) on nonface natural images. We suspect that the function of a region will only be fully understood by considering task-relevant variance across several tasks in light of activity in connected brain regions. The tight interface that we champion between computational models and brain activity should prove useful in evaluating theoretical accounts of how the brain solves tasks over time.

Computational models that perform the tasks end to end, from stimulus to behavior, should be particularly useful. In essence, translating between brain regions to layers of these models can make clear what role a brain region plays within the overall computation. In the case of object recognition, our results suggested that recurrent models may be best positioned to explain how the nature of information within brain regions changes as the computation unfolds.

This conclusion is in line with a growing body of modeling work in neuroscience that affirms the value of recurrent computation (*19*–*21*). Unlike the aforementioned work, we suggest that long-distance recurrent connections that link disparate layers should be considered [cf. (*22*)]. We suspect that such models will be necessary to capture time course data and the duality found in some brain regions, namely, how most variance in a brain region can be attributable to lower-level stimulus properties while co-mingling with important higher-level, task-relevant signals.

As deep learning accounts in neuroscience are extended to other domains, such as audition (*23*) and language processing (*24*), the lessons learned here may apply. Our brain-model interface approach can help evaluate whether the brain processes signals across domains in an analogous fashion. By minding the distinction between shared and task-relevant variance (i.e., activity that can drive the computation), the role that the brain regions play within the overall computation may more readily come into focus.

Our approach may also have practical application in brain machine interfaces (BMIs). Recent BMI developments have emphasized the readout of motor commands, neural processes taking place close to the periphery. In contrast, by leveraging the constraints provided by a pretrained DCNN, we were able to gain traction on the “stuff of thought,” categorical and conceptual information in IT. Because we learned a general translation from brain to model, our approach applied to BMI would allow distant generalization. For example, we were able to extrapolate to novel categories (fig. S3). For example, a translation from the brain to the model that never trained on horses but trained on other categories can perform zero-shot generalization when given brain activity elicited by an image of a horse. The interface has the potential to produce a domain-general mapping rather than one dependent on specific training data. In the future, BMI approaches that address general thought without exhaustive training on all key elements and their combinations may be feasible.

## MATERIALS AND METHODS

### Datasets

We reanalyzed three existing neural datasets. Two, BOLD5000 (*15*) and Generic Object Decoding (*16*), consist of fMRI from human participants who viewed images taken from ImageNet (*25*), a benchmark large dataset of natural images. We restricted the BOLD5000 dataset to only those images drawn from ImageNet (2012 ImageNet Large Scale Visual Recognition Challenge) edition and to participants 1 to 3 who completed the full experiment. The analysis of Generic Object Decoding used the data from the “training” portion of their image presentation experiment, consisting of 1200 images from 150 categories drawn from the ImageNet Fall 2011 edition. For both datasets, each image was presented once; thus, each row represents individual trials.

The third dataset consists of neuron spike counts directly recorded from V4 and IT of two macaque monkeys (*17*) in a rapid serial visual presentation paradigm, where each image is passively viewed for 100 ms, with 100 ms between images. We used the publicly available data processed as detailed in those publications. For the neural interfacing analysis of the spiking neural dataset, we used spike rates aggregated over multiple presentations of each of 3200 unique images, in the interval of 70 to 170 ms after stimulus onset, with the electrodes from the two participants concatenated, as in the original analysis (*18*). For the Granger causal modeling analysis of the same dataset, we used spike rates at the level of the individual trial (i.e., no aggregation) for each 10-ms time bin.

The neural data corresponding with each image were related to layer activations of a DCNN trained on image classification, when processing the same pixel-level data. The three neural datasets contain data for various brain regions from ventral stream, including visual areas [V1, V2, V3, and V4, included as “EarlyVis” in (*15*)], areas responsible for processing shape and conceptual information [lateral occipital complex (LOC) and IT], and various downstream areas [occipital place area (OPA), parahippocampal place area (PPA), FFA, and retrosplenial cortex (RSC)].

For details on neuroanatomical placement or functional localization of each region, we refer the readers to the original publications. Further details of brain regions and dimensionality of the data from each region are presented in table S1.

### Deep convolutional neural network

As the base DCNN for all simulations, we used a reimplemented and trained version of VGG-16 [(*14*); configuration *D*] using Keras (*26*) version 2.2.4 and TensorFlow version 1.12. This model was selected for its uncomplicated architecture, near–human-level classification accuracy on ImageNet, and widely reported robust correspondence with primate or human data on various measures, including human behavioral [similarity judgments (*27*), human image matching (*21*)], and neural (*22*, *28*). We implemented and trained a version of the architecture with an input size of 64 × 64 × 3, with corresponding changes in spatial dimensions for all layers (table S2). For all analyses, images from all datasets were cropped to a square and resized to this resolution. For the monkey multiunit dataset, where images are contained in a circular frame, the central 192 × 192 portion of the 256 × 256 original was cropped and resized, to decrease the proportion of image taken up by blank space in the corners. While the original authors trained their network in a two-stage process, beginning with a subset of the layers, the inclusion of batch normalization (*29*) between the convolution operation and activation function of each layer enabled training the complete network in a single pass. We used the authors’ setting for weight decay (ℓ_2_ penalty coefficient of 5 × 10^−4^) and a slightly different value for dropout probability (0.4). Model architecture details are presented in table S2.

### DCNN training

Our training procedure followed (*14*). The model was trained on ImageNet 2012 (1000 classes) for analyses of the BOLD5000 and monkey multiunit datasets. For the Generic Object Decoding dataset, the model was trained until convergence on ImageNet Fall 2011 (21,841 classes), before layer FC3 was replaced and retrained with 150 classes, corresponding with the classes used in our reanalysis of (*16*). For ImageNet Fall 2011, we randomly allocated 2% of each class including all images used in (*16*) to an in-house validation set that was not used for training. One image used in the original study was missing from our image dataset and was excluded from all analyses. All images were resampled from their native resolution to 64 × 64 × 3 by rescaling the shortest side of the image to 64 pixels and by center cropping.

Both versions of the model were trained using mini-batch stochastic gradient descent, with a batch size of 64, an initial learning rate of 0.001, and a Nesterov momentum of 0.90561. The learning rate decayed by a factor of 0.5 when validation loss did not improve for 4 epochs, with training terminating after 10 epochs of no improvement. All layers used Glorot normal initialization. During training, images were augmented with random rescaling, horizontal flips, and translations. Final accuracy on the respective test sets for each version of the model is provided in table S3.

### Cross-validation

Classifier-based methods require training classifier parameters, before evaluating it on data withheld from the training set. In all analyses, we use the standard approach of *k*-fold cross-validation (*30*), in which the dataset is randomly allocated into *k* equally sized partitions, and the analysis is iterated *k* times, each time training on *k* − 1 partitions and evaluating on one. In this way, the classifier is evaluated over the entire dataset. For all analyses, except where otherwise specified, we use stratified eightfold cross-validation, that is to say, dataset items are randomly allocated to partitions with the constraint that 1/*k* of each class is allocated to each validation partition. For the spiking neural dataset (*17*), each unique image was rendered from 1 of 64 objects, with varying position and orientation. Here, stratification was done at the object level.

For the out-of-training-class generalization analysis, we used leave-one-class-out cross-validation, where for *m* classes, the analysis is iterated *m* times, the evaluation set consisting only of the entirety of a single class, on each iteration.

### Neural interfacing analysis

Given a dataset *D*, consisting of an image matrix *D_i_* of shape (*n*,64,64,3), where *n* is the number of images, and a corresponding neural data matrix *D_r_* of shape (*n*, *d*), where *d* is the number of neural features (electrodes, for multiunit data, or voxels, for fMRI data), consider a DCNN computing a function *f* on *D_i_*, mapping *D* to *P_i_*, an (*n*, *m*) matrix of predictions, each row being a probability distribution over the *m* classes the DCNN was originally trained to classify

$$f(D_{i})=P_{i}$$ (1)

For an arbitrary intermediate model layer *q*, we may decompose *f* into *g_q_* and $g_{q}^{\prime }$, by computing intermediate activations, *g_q_*(*D_i_*)

$$f(D_{i})\equiv g_{q}^{\prime }(g_{q}(D_{i}))=P_{i}$$ (2)

The neural interface analyses proceeded by applying a linear transform *W* to the centered and column-normalized neural data, $D_{r}$, and inputting the result into DCNN layer *q*, to compute a matrix of model predictions for the neural data, $P_{r}$

$$g_{q}^{\prime }(WD_{r})=P_{r}$$ (3)

The transformation matrix *W* was computed by partitioning image and neural datasets *D_i_*, *D_r_* into training and evaluation partitions using 8-fold cross-validation, and *W* was learned as a linear mapping from *D_r_* to the layer *q* activations generated by the corresponding images, *D_i_*, on the training partition

$$g_{q}(D_{i})=WD_{r}+\epsilon$$ (4)

For each cross-validation fold, the model predictions were computed for the evaluation partition. In practice, *W* was computed as a single-layer linear neural network with no bias or activation function, to minimize mean-squared error of supervision targets *g_q_*(*D_i_*) using mini-batch stochastic gradient descent with momentum (batch size of 64, momentum of 0.9, ℓ_2_ regularization of 0.0003, initial learning rate of 0.1, decreasing by a factor of 0.5 when validation loss did not improve for 4 epochs and terminating after 400 epochs or after validation loss did not improve for 20 epochs). For the analysis of the macaque dataset (*16*), on the level of the individual trial, before performing the Granger causality model, *W* was computed using the Adadelta optimizer (batch size of 128 and initial learning rate of 0.04).

We also considered an alternative mode for training *W*, by first assembling the model in the form of Eq. 3, composed of transformation matrix *W* initialized with small random weights, followed by DCNN layer *q* onward, *g_q_*′, thus mapping end to end from neural measures *D_r_* to output. *W* was then trained by back-propagating the categorical cross-entropy error term from the softmax output layer, using the supervision target of the ground-truth labels for the neural dataset (*D_r_*), with all other weights in the network frozen. This method produced a pattern of results that were qualitatively similar, although with lower absolute accuracy (fig. S4).

Given the success of dimensionality reduction techniques and penalized regression models in shared variance analyses (*31*), we explored those techniques in training the linear transformation matrix. Specifically, we computed an intermediate latent space by computing the first 5000 principal components of model layer *q* activations (trained on activations from 30,000 images randomly sampled from the ImageNet 2012 training set) and using this model to reduce the target activations, *g*_q_(*D*_i_), to 5000 dimensions. Learning the linear transformation matrix to this lower-dimensional space proceeded as above, with varying levels of ℓ_2_ regularization. The results (fig. S5) show the same pattern of findings as previously described, with lower absolute accuracy. Better results are obtained with no ℓ_2_ penalty.

#### Neural interface evaluation

The output of the model, *P*, is an (*n*, *m*) matrix of probability distributions over the *m* output classes where the original DCNN was trained on, for each of *n* images in *D*. We computed this for the original DCNN on the image dataset, *f*(*D_i_*) = *P_i_*, and also for the neural dataset for each brain region *r* and model layer *q*, $g_{q}^{\prime }(WD_{r})=P_{r}$. The correspondence between *r* and *q* was evaluated by comparing the model predictions *P_r_* either against model predictions from the image dataset [by computing the KL divergence of *P_r_* from *P_i_* for each row *n*) or against the ground-truth classes (by computing the overall AUC (area under the receiver-operator characteristic curve) of the classifier]. The AUC of the classifier was calculated as a multiclass generalization of the two-class AUC statistic by averaging overall pairs of classes (*32*). Each pairwise AUC was calculated via its equivalence with the Wilcoxon-Mann-Whitney *U* statistic (*33*): For each pair of classes (*X*, *Y*), *U* is calculated as the proportion overall *n* instances of *X* and *m* instances of *Y*, and the number of cases where *X* is assigned a higher probability than *Y*

$$U=\sum_{i=1}^{n}\sum_{j=1}^{m}S(X_{i},Y_{j})$$ (5)

where

$$S(X,Y)=\{\begin{array}{c} 1\;\text{if}\;X>Y\\ 0\;\text{if}\;X<Y\\ \frac{1}{2}\;\text{if}\;X=Y \end{array}$$ (6)

#### Interfacing pixel-level data

For comparison, we conducted the interfacing analysis mapping pixel-level data derived from the images themselves in place of neural data. The BOLD5000 image stimuli, sized at 64 × 64 × 3 pixels, were flattened and projected down to the first 1200 principal components using a principal components analysis (PCA) model trained on 20,000 images randomly sampled from the ImageNet 2012 training set. These 1200-dimensional image representations were used in place of neural data, with the remainder of the analysis proceeding identically with an adjusted learning rate of 0.4. Results (fig. S6) show best accuracy when interfaced with the earliest layers of the model (Conv1b) and demonstrate that interfacing with earliest model layers produces better results than late layers, provided that there are sufficient data.

### Shared neural variance analysis

For comparison, we present an example of a shared neural variance analysis using the macaque spiking neuron dataset (*17*) and our reimplemented model. Conceptually, in common with the interfacing analysis (Fig. 2), the analysis evaluates the correspondence between a brain region *r* and a model layer *q*. Layer *q* model activations, *g_q_*(*D_i_*), were compared with a neural dataset obtained from the presentation of corresponding images, *D_r_*. To establish that our results are comparable to those of the previous study, we used the neural predictivity method exactly as implemented in the Brain-Score benchmark for DCNNs (*33*).

The dataset was iteratively partitioned using 8-fold cross-validation into the training/validation partitions. Following the method in (*24*), we used the image stimuli from the training partition to generate model activations on each layer. We used PCA to calculate the first 1000 principal components of these activations, before training a partial least squares regression model (25 components) to predict, for each electrode, the firing rate across the validation partition. The predictivity for each electrode was computed as the Pearson correlation coefficient between the predicted firing rates across the dataset and the actual recorded values, with the overall predictivity given by the correlation coefficient of the median electrode.

### Simple classifiers on the neural datasets

To establish performance baselines for the interfaced fMRI datasets, which were evaluated in terms of classification performance, we applied various standard classifiers to the neural data directly, to predict the image class from the neural data from various brain regions. Known as MVPA, evaluating the trained classifier’s ability to predict class labels from fMRI or spiking neural data is now a standard approach to quantifying the categorical-level information within a brain region (*34*). Nevertheless, in the present analyses, the number of different classes is unusually large, and the number of examples from each class is unusually small [1916 images from 958 classes (*15*) and 1200 images from 150 classes (*16*)] for a straightforward MVPA analysis on these datasets. We report the AUC of the classifier computed in the same way as for the neural interfacing analysis. All classifiers were implemented as detailed below using version 0.20.3 of the scikit-learn library (*35*).

#### Multiclass logistic regression

The classifier was implemented as LogisticRegression with the “multinomial” option, the lbfgs solver, and a maximum of 10^3^ iterations.

#### Nearest-neighbor classifier

The classifier was implemented as KNeighborsClassifier. Given the structure of the BOLD5000 dataset, with only two examples per class (thus, either one or two examples in the training partition, test classification of each class on the basis of one correct training example), we classified on the basis of the single nearest neighbor under a Euclidean distance function.

#### Linear support vector machine

The classifier was implemented as LinearSVC, using a one-versus-rest multiclass strategy, with a maximum of 10^4^. iterations and *C* parameter of 10^−3^

### Granger causal modeling

In contrast to the previous neural interfacing analysis of the spiking neural dataset, which aggregated spike rates over multiple presentations of each image, in the interval of 70 to 170 ms after stimulus onset, here, we trained and evaluated the model on data at the individual trial level. We conducted a separate decoding analysis for each 10-ms time bin, from −20 ms (i.e., before stimulus onset) to 270 ms, with all time indices referring to the preceding 10-ms time bin. Training linear transformation matrix *W* is described in the Neural Interfacing Analysis section of the method. Before the GCM, we preprocessed the trial-level relative entropy data to ensure stationarity by, first, subtracting the temporal mean and SD from each trial and, second, subtracting the mean signal and dividing by the signal’s SD, thus ensuring that each time step has zero mean and unit variance.

Given two regions, *X* and *Y*, separate Granger causal models were computed for each direction *X* → *Y* and *X* ← *Y*, where each model takes the form of a linear regression, where the univariate outcome

$$\text{KL}{\left(D_{X}\|D_{i}\right)}_{n}$$ (7)

where the KL divergence of region *X* with θ, the base model predictions, is predicted by the Granger null model (8) or the Granger causal model (9)

$$\text{KL}{\left(D_{X}\|D_{i}\right)}_{n-1},\ldots ,\text{KL}{\left(D_{X}\|D_{i}\right)}_{n-p}$$ (8)

$$\text{KL}{\left(D_{X}\|D_{i}\right)}_{n-1},\text{KL}{\left(D_{Y}\|D_{i}\right)}_{n-1},\ldots ,\text{KL}{\left(D_{X}\|D_{i}\right)}_{n-p},\text{KL}{\left(D_{Y}\|D_{i}\right)}_{n-p}$$ (9)

where *p*, the maximum number of previous time steps, is a hyperparameter that is determined using model selection criteria such as Bayesian Information Critereon. The appropriate model was determined by comparing log-likelihood ratios, given the data, for the causal and null models.

### Perturbation analysis

One possible explanation for the observed pattern of neural interfacing results (Fig. 2) is the prediction error in input model activations becoming compounded over subsequent layers of DCNN processing in the DCNN. This may occur if perturbations in DCNN activations on a given layer become larger on subsequent layers. The opposite case is that DCNN layers implement a tolerance to activation error, such that perturbations become smaller on subsequent layers. We conducted a perturbation analysis (fig. S7), in which the BOLD5000 images were input to the base DCNN and activations on a given layer were perturbed with Gaussian noise (SD of γσ, where σ is the SD of each unit’s activations and γ is a scaling parameter equal to 4.0), and the downstream effects on the final convolutional layer (Conv5c) were compared with those resulting from nonperturbed activations. The results show that perturbations on the preceding convolutional layer (Conv5b) produced much greater error on Conv5c than on layers further upstream. These results suggest that subsequent DCNN layers effectively correct activation error occurring upstream. We take these findings as suggestive that the prediction error does not bias neural decoder accuracy against early layers.

## Notes

View/request a protocol for this paper from *Bio-protocol*.
