# Supplementary material for: Reassessing hierarchical correspondences between brain and deep networks through direct interface
Source: Sci Adv. 2022 Jul 13;8(28):eabm2219. doi: 10.1126/sciadv.abm2219 (PMC9278854; doi:10.1126/sciadv.abm2219)
Supplement: Supplementary file 1 — Figs. S1 to S10 Tables S1 to S3 [file sciadv.abm2219_sm.pdf]

Science Advances
AAAS

Supplementary Materials for

Reassessing hierarchical correspondences between brain and deep networks through direct interface

Nicholas J. Sexton and Bradley C. Love

Corresponding author: Nicholas J. Sexton, n.sexton@ucl.ac.uk

Sci. Adv. **8**, eabm2219 (2022)
DOI: 10.1126/sciadv.abm2219

This PDF file includes:

Figs. S1 to S10
Tables S1 to S3

SI Figures

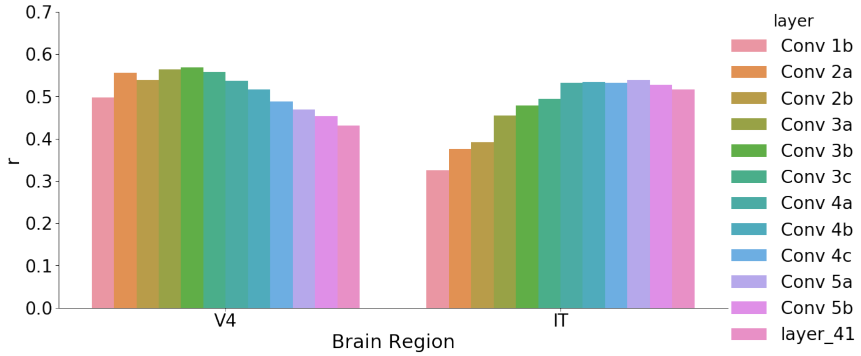

Figure S1: Standard approaches to relating primate ventral stream and DCNNs evaluate variance shared between data from each brain region and unit activations on each DCNN layer. They have been taken as evidence that earlier ventral stream regions (e.g., V4) correspond to earlier DCNN layers and later regions (e.g., IT) correspond to later DCNN layers. Here, we present a shared-variance based analyses of directly recorded spiking neural activity (*16*) and VGG-16 using an established method (*20*) Higher correlations reflect more shared variance between brain region and model layer.

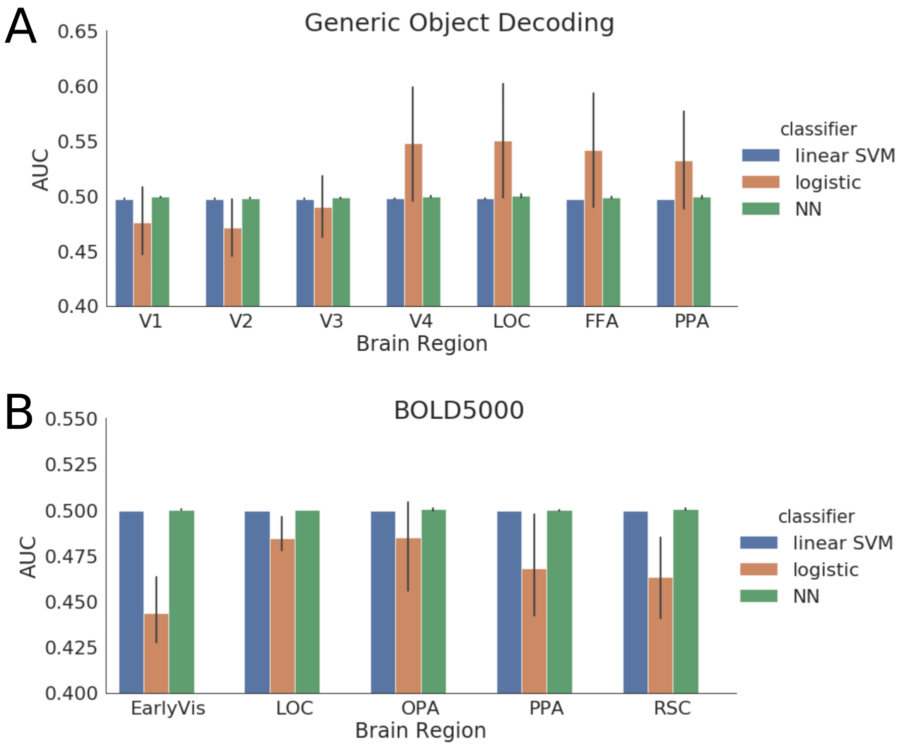

Figure S2: Comparison of the brain-DCNN interface as a neural pattern classifier, compared with standard linear classifiers typically used in multi-voxel pattern analysis (MVPA). We present classification performance (AUC) directly on neural patterns, on the Generic Object Decoding (**A**) and BOLD5000 (**B**) datasets, for simple classifiers (support vector machine with linear kernel, multiclass logistic regression, and a 1-nearest neighbour classifier) with results for interface with each layer of the DCNN presented for comparison. Performance of the simple classifiers is generally near chance (0.5), we attribute this to the large number of image classes (150, 958 respectively) and few available examples (2, 8 per class) which severely limit the available training data. Because the brain-DCNN interface learns a general mapping between brain region and model, it does not suffer this limitation, making it an appealing novel approach for MVPA.

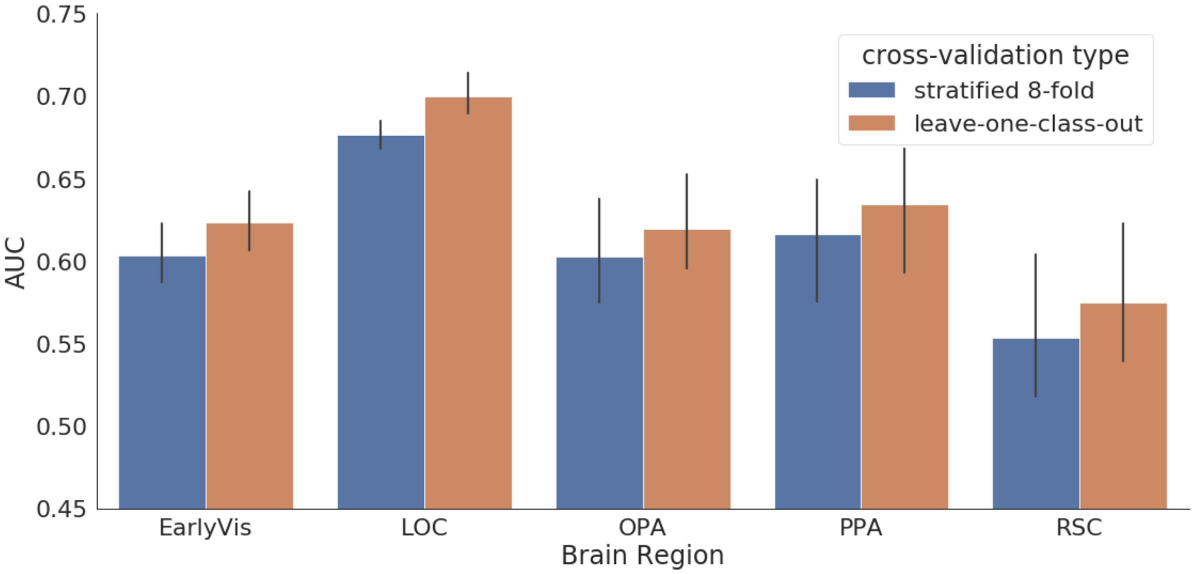

Figure S3: Learning a mapping directly from neural measures to DCNN activation space produces a general mapping, rather than being dependent on training examples. The neural interface has an in-built ability to generalise to novel classes. This is demonstrated by presenting classification performance (AUC) on the BOLD5000 dataset, by comparing cross-validation (CV) strategies. Error bars represent 95% confidence intervals across 3 subjects. Stratified 8-fold CV (the default, used in all other analysis) ensures each training partition contains at least one example of each class. Leave-one-class-out CV involves the same number of CV folds as there are classes, each time training on all data except one class, which is withheld for the validation set. Performance is equivalent or better (LOC) when generalising to novel classes, which we attribute to more training data per CV fold. Due to the training time, this analysis was restricted to layer 5a.

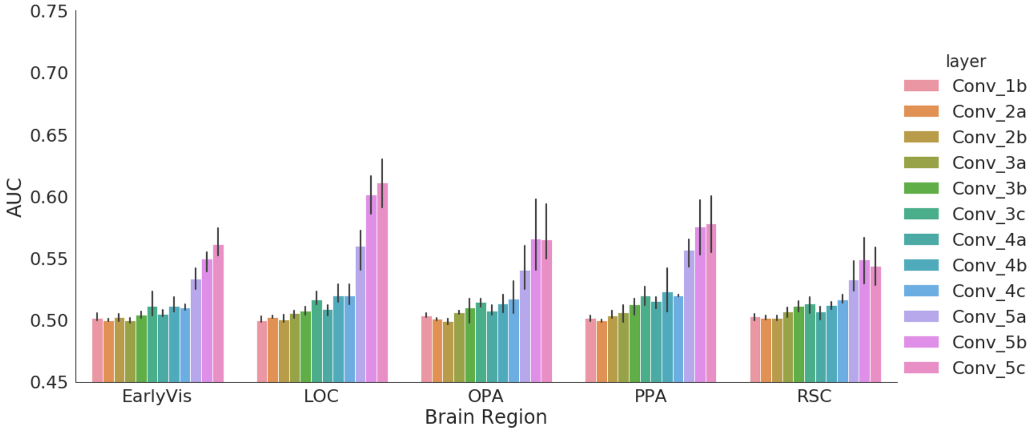

Figure S4: Alternative 'backprop mode' for training the transformation matrix W mapping from neural space to DCNN activation space. Classification performance (AUC) on the BOLD5000 dataset follows a qualitatively similar pattern to the main analysis (compare fig. 2B), albeit with lower absolute accuracy. The default analysis trains W independently as a regression problem, using layer activations as supervision targets directly. Instead, this approach uses W as a weights matrix for a new neural network that takes neural data from a brain region as input, connected to the latter part of the DCNN, and training the network using the class labels as supervision targets, with all other DCNN weights frozen.

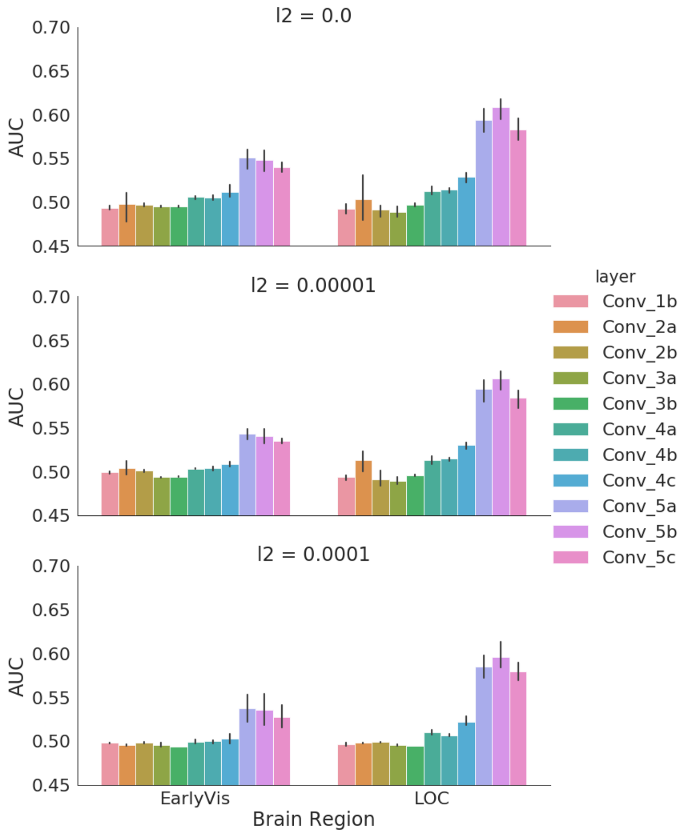

Figure S5: Alternative procedure for training linear mapping. Model activations on the BOLD5000 dataset are projected down to first 5000 principal components, with the linear map trained to predict in this reduced-dimensionality latent space, before being projected back up to native dimensionality for each layer. Analyses repeated for different levels of ℓ_2 regularisation. Results show a similar pattern to the main analysis, with lower absolute accuracy. Best results are obtained with zero ℓ_2 penalty.

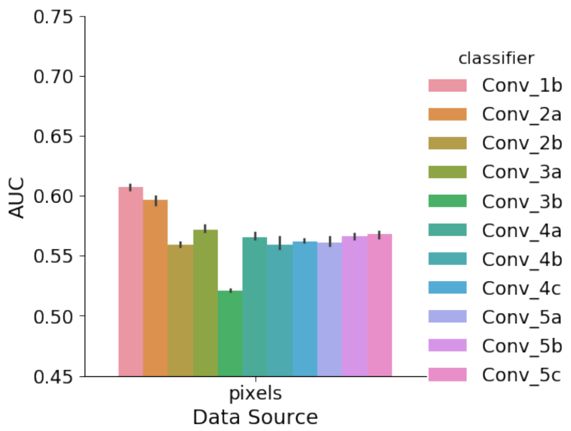

Figure S6: Interfacing pixel-level data from BOLD5000 stimulus images with DCNN in place of neural data. These results demonstrate correspondence of early layers with early layer-like information.

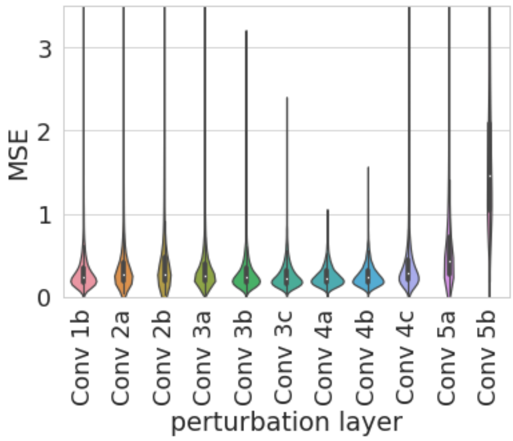

Figure S7: Perturbation analysis. Base model activations on each layer were perturbed by adding Gaussian noise, with MSE of unit activations on a downstream layer (Conv5c) evaluated against those evoked by nonperturbed activations. The distribution of Conv5c MSE across the BOLD5000 image stimulus dataset is plotted for each layer. These results suggest the greatest downstream effect comes from perturbing the immediately preceding layer's activations, with perturbations further upstream having a minor effect.

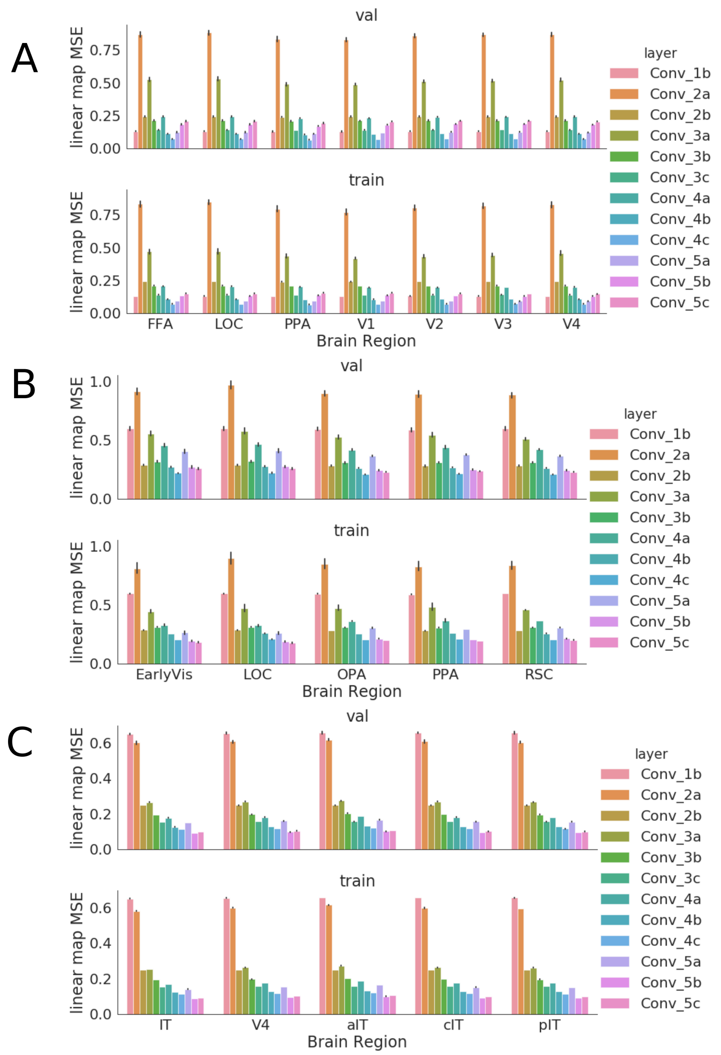

Figure S8: Fit (mean squared error) of the linear projection from brain data to DCNN activations on each layer, for training and evaluation phases. (**A**) Generic Object Decoding (**B**) BOLD5000 (**C**) Linear Weighted Sums. Note that MSE is affected by dimensionality and scaling based on learned weights on each layer, therefore may not be directly comparable across layers.

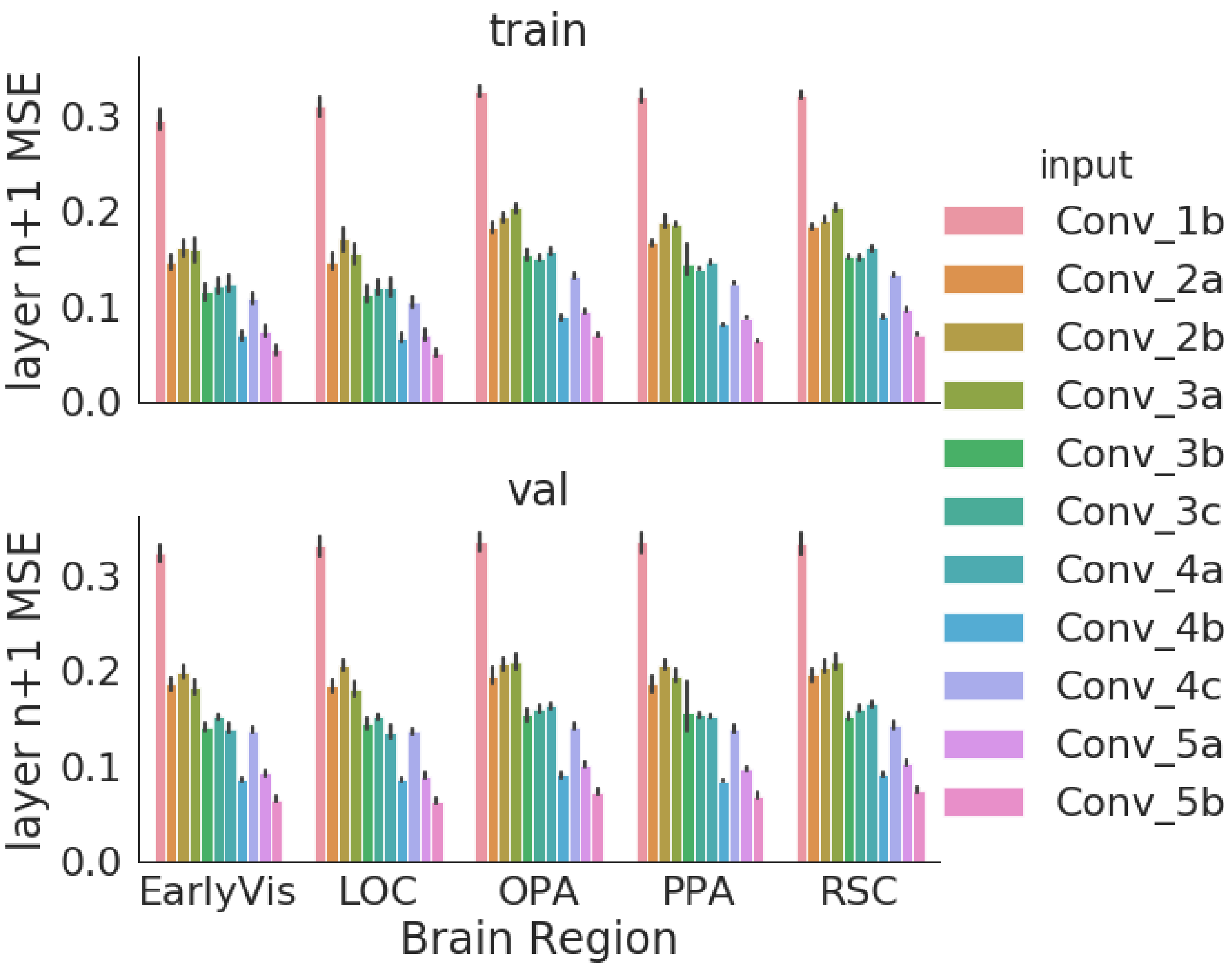

Figure S9: Downstream error: For neural data input into model layer n, the figure shows the distance (MSE) in model activations computed on the n+1 convolutional layer, compared with those evoked by image-input on the BOLD5000 dataset. Note that, confirming results from the perturbation analysis (S7), downstream error is smaller than the linear map error (S8).

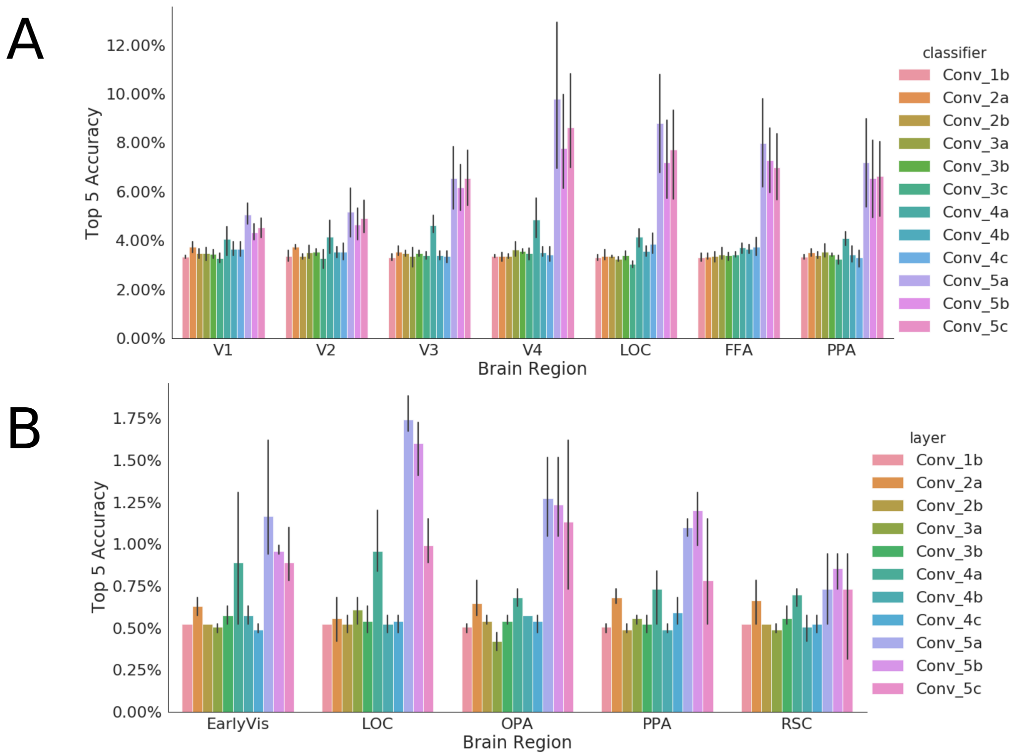

Figure S10: Top 5 accuracy of predictions from brain data (**A**: Generic Object Decoding, **B**: BOLD5000) showing a similar pattern to evaluation using AUC. Note that for the number of categories (958 and 150 categories respectively), corresponding chance accuracy levels are 0.52% and 3.3%.

SI Tables

Dataset	Generic Object Decoding(*16*)	BOLD5000(*15*)	Linear Weighted Sums(*17*)
Stimuli	experiment 'train' phase: 1200 images from 150 categories (ImageNet Fall 2011)	1916 images from 958 categories (ImageNet ILSVRC 2012	3200 greyscale composite images, 64 objects in 8 categories,) non-congruent background
Task	one-back repetition detection	valence judgement ('like', 'neutral', 'dislike')	passive viewing, RSVP presentation 100ms/100ms
Subjects	5 human fMRI	3 human fMRI (partial data from subject 4 excluded)	2 Macaque monkeys (vectors concatenated) multi-unit recording
Time indices	full 9s of image presentation	TR3-4	70-170ms
Brain region (dimensionality per subject)	V1 (1004, 757, 872, 719, 659)<br>V2 (1018, 944, 1031, 855, 891)<br>V3 (759, 810, 861, 929, 907)<br>V4 (740, 544, 754, 704, 860)<br>LOC (540, 834, 996, 668, 566)<br>PPA (356, 316, 496, 398, 550)<br>FFA (568, 435, 928, 725, 929)	EarlyVis (495, 495, 1218)<br>LOC (342, 888, 1027)<br>OPA (288, 180, 392)<br>PPA (331, 370, 273)<br>RSC (229, 421, 394)	IT (168 = 58 + 110)<br>V4 (88 = 70 + 18)
Stimulus field of view	12°	4.6°	8° (6° after cropping)

Table S1: Neural datasets For further dataset details, such as how regions were defined, we refer readers to the original publications

Block	Layer	Dimensions ($h \times w \times c$)	Filter Size
Input		$64 \times 64 \times 3$	
1	1a	$64 \times 64 \times 64$	3×3
	1b	$64 \times 64 \times 64$	3×3
	max pool 1		2×2
2	2a	$32 \times 32 \times 128$	3×3
	2b	$32 \times 32 \times 128$	3×3
	max pool 2		2×2
3	3a	$16 \times 16 \times 256$	3×3
	3b	$16 \times 16 \times 256$	3×3
	3c	$16 \times 16 \times 256$	3×3
	max pool 3		2×2
4	4a	$8 \times 8 \times 512$	3×3
	4b	$8 \times 8 \times 512$	3×3
	4c	$8 \times 8 \times 512$	3×3
	max pool 4		2×2
5	5a	$4 \times 4 \times 512$	3×3
	5b	$4 \times 4 \times 512$	3×3
	5c	$4 \times 4 \times 512$	3×3
	max pool 5		2×2
FC	FC1	4096	
	dropout 1		
	FC2	4096	
	dropout 2		
	FC3 (output)	1000 softmax	

Table S2: DCNN Architecture: Layer configuration and dimensions of the DCNN used for all analyses.

Training Set	Top 1 Accuracy	Top 5 Accuracy
Imagenet ILSVRC 2012 (1000 classes)	57.64%	80.53%
Imagenet Fall 2011 (21841 classes)	37.74%	62.51%

Table S3: DCNN Performance. Base DCNN (64x64) accuracy on training datasets.
